# Exploring the Relationship Between Ejection Fraction, Arterial Stiffness, NT-proBNP, and Hospitalization Risk in Heart Failure Patients

**DOI:** 10.3390/diagnostics14242885

**Published:** 2024-12-22

**Authors:** Gyongyi Osser, Brigitte Osser, Csongor Toth, Caius Calin Miuța, Gabriel Roberto Marconi, Laura Ioana Bondar

**Affiliations:** 1 Faculty of Physical Education and Sport, “Aurel Vlaicu” University of Arad, 310130 Arad, Romania; gyongyi.osser@uav.ro (G.O.); brigitte.osser@uav.ro (B.O.); csongor.toth@uav.ro (C.T.); gabriel.marconi@uav.ro (G.R.M.); 2 Doctoral School of Biomedical Sciences, University of Oradea, 410087 Oradea, Romania; bondar.lauraioana@student.uoradea.ro; 3 Department of Biology and Life Sciences, “Vasile Goldiș” Western University of Arad, 310048 Arad, Romania

**Keywords:** augmentation index, arterial stiffness, cardiovascular health, ejection fraction, heart failure, hospitalization risk, pulse wave velocity

## Abstract

**Background/Objectives:** Heart failure (HF) remains a leading cause of hospitalization and morbidity. Arterial stiffness, measured by pulse wave velocity (PWV) and the augmentation index (AIx), has been linked to HF severity and prognosis. This study investigates the relationship between clinical parameters, biochemical indicators, and arterial stiffness in hospitalized patients with HF, aiming to identify predictors of hospitalization and improve patient management. **Methods:** This cross-sectional study included 98 patients admitted with HF: 53 with acutely decompensated HF (sudden worsening of symptoms) and 45 with chronic HF (stable symptoms of HF). Clinical and biochemical parameters, including ejection fraction (EF), N-terminal prohormone of brain natriuretic peptide (NT-proBNP) levels, and arterial stiffness indicators (PWV and AIx), were measured at admission. During follow-up, 59 patients required re-hospitalization due to acutely decompensated HF, while 39 remained outpatients without further hospitalization. The relationship between these parameters was analyzed using Pearson correlation coefficients, and multiple Cox regression analysis was conducted to identify independent predictors of re-hospitalization. **Results:** A significant negative correlation between EF and PWV was found (r = −0.853, 95% CI [−0.910, −0.764]), suggesting an association between improved heart function (higher EF) and reduced arterial stiffness (lower PWV). A moderate positive correlation between EF and AIx (r = 0.626, 95% CI [0.473, 0.805]) suggests that, while higher EF is associated with increased AIx, the relationship is weaker compared to EF and PWV. This may reflect differing contributions of vascular and myocardial factors to HF severity. Hospitalized patients exhibited significantly poorer clinical and biochemical profiles, including higher NT-proBNP levels (*p* < 0.001) and worse blood pressure (BP) measurements (systolic and diastolic, *p* < 0.01). Multiple Cox regression analysis identified PWV, Aix, and NT-proBNP as independent predictors of re-hospitalization in HF patients, with significant hazard ratios: PWV (HR = 1.15, *p* = 0.02), AIx (HR = 1.03, *p* = 0.02), and NT-proBNP (HR = 1.0001, *p* < 0.01). **Conclusions:** Arterial stiffness indices (PWV and AIx), EF, and NT-proBNP were identified as significant predictors of re-hospitalization in HF patients. These findings suggest that integrating arterial stiffness measurements into routine clinical assessments may enhance the risk stratification and inform targeted interventions to reduce hospitalizations and improve outcomes.

## 1. Introduction

Heart failure (HF) represents a significant global health challenge, characterized by the heart’s inability to pump blood effectively, leading to symptoms such as fatigue, shortness of breath, and fluid retention [1,2]. The World Health Organization estimates that approximately 64 million people are living with HF worldwide, with the rising prevalence attributed to an aging population and increasing rates of hypertension (HTN), diabetes mellitus (DM), and coronary artery disease [3,4]. HF results from the heart’s inability to maintain adequate cardiac output to meet the body’s demands, which can be due to either impaired systolic function (heart’s pumping ability) or diastolic dysfunction (inability of the heart to fill properly). Over time, this leads to maladaptive changes in the heart and vasculature, including ventricular dilation, myocardial fibrosis, and increased afterload, all of which worsen the condition [5,6]. 

Patients with HF frequently experience episodes of acutely decompensated HF, which can lead to hospitalization and are associated with high morbidity and mortality rates [7,8]. Decompensation refers to the worsening of HF symptoms, often due to factors such as non-compliance with medication, increased salt intake, infections, or worsening comorbid conditions. During this acute phase, the compensatory mechanisms, such as activation of the renin–angiotensin–aldosterone system (RAAS) and sympathetic nervous system, become overwhelmed, leading to fluid retention, vasoconstriction, and further deterioration in cardiac output. This cascade of pathophysiological events exacerbates congestion and organ dysfunction, requiring increased medical intervention, hospitalization, or a significant decline in functional status [9,10]. Understanding the clinical parameters and risk factors that contribute to these adverse outcomes is critical to improve patient management and treatment strategies [11,12]. 

Traditionally, the assessment of the ejection fraction (EF) has been the cornerstone of the evaluation of HF, serving as a key indicator of cardiac function [13,14]. EF measures the percentage of blood pumped out of the left ventricle with each contraction, with reduced EF typically reflecting systolic dysfunction [15,16]. However, recent research has underscored the importance of biomarkers such as N-terminal pro b-type Natriuretic Peptide (NT-proBNP), as well as measures of arterial stiffness, including pulse wave velocity (PWV) and augmentation index (AIx), in predicting hospitalizations and mortality risk among HF patients [17,18]. These biomarkers reflect underlying pathophysiological processes that contribute to poor outcomes in HF patients, such as neurohormonal activation, vascular remodeling, and endothelial dysfunction [19,20]. Studies suggest that lower EF correlates with higher arterial stiffness, but the interplay between these indicators and their combined predictive power remains an area of active investigation [21,22,23].

Despite well-established associations between these clinical parameters and HF outcomes, a more nuanced understanding of their interactions is needed, especially in the context of acutely decompensated HF [24]. Some researchers advocate for the integration of arterial stiffness assessments to enhance risk stratification and therapeutic decision-making, positing that traditional measures may not fully capture the complexity of HF [25,26]. Arterial stiffness, for instance, represents a form of vascular remodeling that occurs due to increased afterload and systemic inflammation, further complicating the management of HF [27,28]. Additionally, controversy regarding the relevance of gender differences in HF presentations and outcomes suggests potential biases in treatment approaches, highlighting the unique needs of different patient populations [29,30].

The main objective of this study is to investigate the relationship between clinical and biochemical parameters, including EF, NT-proBNP, and arterial stiffness indicators, in hospitalized patients with acutely decompensated HF. By elucidating the predictors of hospitalization risk and overall clinical status, this research seeks to identify key indicators that can guide therapeutic strategies.

The rationale of this study is to address critical gaps in the existing literature regarding HF management. By focusing on the interplay between traditional clinical metrics, such as the EF and N-terminal prohormone of brain natriuretic peptide (NT-proBNP) levels, and emerging indicators of arterial stiffness, including PWV and AIx, this study aims to enhance the risk stratification and treatment outcomes for high-risk populations. Understanding how these factors interact will contribute to identifying key predictors of hospitalization and the overall clinical status in patients with acutely decompensated HF. Ultimately, the findings may provide insights that lead to more targeted interventions, improved management strategies, and enhanced patient outcomes in HF care.

## 2. Materials and Methods

### 2.1. Study Design and Setting

This study is a cross-sectional analysis conducted from November 2022 to October 2024 in the Department of Cardiology, Arad Clinical Emergency Hospital, Romania. The aim of this study is to evaluate clinical, biochemical, echocardiographic parameters and arterial stiffness indicators in hospitalized patients diagnosed with acutely decompensated HF.

### 2.2. Study Population

The study included 98 hospitalized patients with HF classified under New York Heart Association (NYHA) classes III–IV and EF ≤ 35%, determined by transthoracic echocardiography (TTE). At admission, patients were categorized into two groups based on clinical status:

- Acutely decompensated HF (*n* = 53): Patients hospitalized due to acute HF exacerbation, such as worsening dyspnea, fluid overload, or acute clinical instability requiring immediate intervention.
- Chronic HF (*n* = 45): Patients hospitalized for other clinical indications, such as routine evaluations or comorbidities, while exhibiting stable HF symptoms.

### 2.3. Inclusion Criteria

Patients meeting the following criteria were included in the study:

- Adult patients
- Patients across both genders
- Patients admitted to the Department of Cardiology, Arad Clinical Emergency Hospital, Romania, and evaluated at least once as part of the study inclusion process
- Patients with a confirmed diagnosis of HF (NYHA III–IV)
- EF ≤ 35%
- Signed informed consent for participation in the study

### 2.4. Exclusion Criteria

Patients not meeting the following criteria were excluded from the study:

- Patients with any of the following conditions were excluded from the study:
  ○ Atrial fibrillation
  ○ Frequent ventricular ectopy
  ○ Advanced renal failure
  ○ Hypothyroidism or hyperthyroidism
  ○ Chronic respiratory diseases (e.g., chronic obstructive pulmonary disease, chronic bronchitis, or pulmonary embolism)
  ○ Primary pulmonary HTN
  ○ Acute coronary syndrome within the last two months
  ○ Congenital heart diseases
  ○ Advanced valvular disease (e.g., mitral stenosis, aortic stenosis, or severe valvular regurgitation)
  ○ Constrictive pericarditis
- Patients without HF classified as NYHA III–IV
- Patients who did not provide informed consent for participation

### 2.5. Diagnosis

Systolic dysfunction was diagnosed using TTE performed with the GE Vivid E95 echocardiographic machine (General Electric, Chicago, IL, USA), employing a 3.5 MHz transducer. EF was calculated using the modified Simpson’s biplane method in accordance with the American Society of Echocardiography (ASE) guidelines. This method involves tracing the endocardial border in apical four-chamber and two-chamber views to derive the left ventricular volumes. Additional parameters, such as ventricular wall thickness and motion abnormalities, were assessed, and a left ventricular end-diastolic diameter (LVEDD) was measured in the parasternal long-axis view. Conversely, diastolic dysfunction was identified in patients with EF > 45% and fractional shortening ≥ 28% in the absence of contractility abnormalities but with altered filling or relaxation patterns, as demonstrated by Doppler examination.

### 2.6. Data Collection

Data collection was structured into three stages:

1. Baseline Data: Collected at admission, including demographics, clinical parameters, and arterial stiffness indicators.
2. Re-hospitalization Events: Documented for patients requiring re-admission due to acutely decompensated HF.
3. Follow-Up Evaluations: Conducted at 3 and 6 months, focusing on clinical outcomes, echocardiographic measures, and arterial stiffness indicators.

Data on the demographic and clinical characteristics, including age, gender, HTN, DM, hyperlipidemia, coronary artery disease, smoking habits, and family history of cardiovascular diseases, were collected for all participants. The following variables were recorded:

- Comorbidities and ongoing treatment
- Laboratory analyses, including hemoglobin (Hb) and serum creatinine levels
- Blood pressure (BP) measurements, conducted in accordance with the European Society of HTN guidelines, with peripheral pulse measured at the radial artery

Arterial stiffness indicators were assessed using PWV and AIx. Measurements were conducted with an oscillometric device, the MedExpert Arteriograph (TensioMed, Budapest, Hungary), a validated system for non-invasive arterial stiffness assessment. PWV was determined by measuring the transit time of pressure waves between the carotid and femoral arteries. The distance between these two sites was measured manually with a tape measure, while the device automatically calculated the PWV based on the detected time delay. AIx was derived from the aortic pressure waveform and expressed as a percentage of the augmentation pressure relative to the pulse pressure. All measurements were performed with patients in the supine position after at least 10 min of rest.

### 2.7. Ethical Considerations

This study was conducted in accordance with the Declaration of Helsinki. Ethical approval was granted by the Ethics Committee of the Arad Clinical Emergency County Hospital in Romania, with approval code 1436/2/16 October 2022. All participants provided informed consent prior to inclusion in the study.

### 2.8. Statistical Analysis

Statistical analyses were conducted using SPSS Statistics 26.0 (IBM Corp., Armonk, NY, USA). Continuous variables are presented as the mean ± standard deviation, while ordinal variables are reported as absolute and relative frequencies. Group comparisons were performed using the Student’s *t*-test for continuous variables and Pearson’s correlation coefficients for continuous variables. The Pearson correlation coefficient was used to assess the strength and direction of the linear relationship between two continuous variables. A positive r-value indicates a direct relationship, where an increase in one variable is associated with an increase in the other, while a negative r-value indicates an inverse relationship. The strength of the relationship is categorized as weak (r between 0 and 0.3), moderate (r between 0.3 and 0.7), or strong (r between 0.7 and 1.0) based on the magnitude of the correlation coefficient. To evaluate predictors of re-hospitalization in HF patients, a multiple Cox regression analysis was performed. Hazard ratios (HRs) with 95% confidence intervals (CIs) were calculated for each variable included in the model. The analysis was adjusted for potential confounders, such as age, sex, and relevant comorbidities. A *p*-value of ≤ 0.05 was considered statistically significant.

### 2.9. Follow-Up Protocol and Study Endpoints

The primary endpoint of this study was hospitalization due to acutely decompensated HF, while the secondary endpoints included changes in the echocardiographic parameters, arterial stiffness indicators (PWV and AIx), and NT-proBNP levels. Clinical data, including EF, arterial stiffness indices, and biomarkers, were collected at the time of admission. Patients were followed up over a period of 6 months, with assessments at 3 and 6 months to monitor changes in clinical status, including re-hospitalization rates and improvements or deterioration in HF symptoms. The primary endpoint was defined as the first episode of acutely decompensated HF requiring re-hospitalization. Patients with chronic HF who did not experience decompensation were re-evaluated clinically, paraclinically, and echocardiographically during scheduled follow-up visits at the Integrated Specialty Outpatient Clinic.

### 2.10. Hypotheses of the Study

This study investigates the complex relationships between various clinical, biochemical, and echocardiographic parameters in hospitalized patients with HF. The aim of this study is to establish how these parameters can predict hospitalization risk and clinical deterioration. To support this investigation, the following hypotheses were formulated based on the current literature and clinical observations:

1. Primary hypothesis: In patients with acutely decompensated HF, there is a significant correlation between EF and indices of arterial stiffness. Specifically, a strong negative correlation between EF and PWV is expected, suggesting that improved cardiac function is associated with reduced arterial stiffness. In contrast, a more moderate positive correlation between EF and AIx may indicate a less pronounced relationship between heart function and arterial stiffness.
2. Comparative hypothesis: Re-hospitalized patients with acutely decompensated HF have significantly worse clinical and biochemical parameters, including elevated NT-proBNP levels, changes in BP levels, and renal dysfunction, compared to non-hospitalized patients.
3. Risk factor hypothesis: The prevalence of cardiovascular risk factors (e.g., HTN, DM, and dyslipidemia) differs significantly between re-hospitalized and non-hospitalized patients, indicating that certain risk factors are associated with acutely decompensated HF.
4. Predictive hypothesis: Indicators of arterial stiffness, specifically PWV and AIx, serve as independent predictors of hospitalization in patients with acutely decompensated HF, even after controlling for other clinical parameters such as age, gender, renal function, and NT-proBNP levels.
5. Gender distribution hypothesis: Significant differences in gender distribution were observed between the chronic and acutely decompensated HF groups. A higher proportion of males was found in the chronic HF group, while females were more prevalent in the acutely decompensated HF group.
6. Comorbidity hypothesis: Patients experiencing acutely decompensated HF will show a higher prevalence of comorbidities compared to those with chronic HF, suggesting that the presence of comorbid illnesses exacerbates HF severity.
7. Re-hospitalization risk hypothesis: In patients with HF, arterial stiffness indices (PWV and AIx), EF, and NT-proBNP levels serve as independent predictors of re-hospitalization. It is hypothesized that these parameters will significantly influence the risk of re-hospitalization, even after adjusting for other clinical variables such as age, gender, and comorbidities, as assessed by multiple Cox regression analysis.

## 3. Results

At the time of admission, all 98 patients were hospitalized, comprising 53 with acutely decompensated HF and 45 with chronic HF. Throughout the follow-up period, 59 patients required re-hospitalization due to HF decompensation, while 39 remained outpatients without further hospitalization.

### 3.1. Correlations Between Clinical, Biochemical, Echocardiographic Parameters, and Arterial Stiffness Indicators in HF Patients

#### 3.1.1. Clinical Characteristics of the Entire Patient Group

The clinical characteristics of the entire cohort (*n* = 98) are summarized in Table 1. The mean age of the cohort was 61.00 ± 6.27 years, with the majority of male patients (71.43%) compared to female patients (28.57%). The mean EF was 27.83% ± 5.22%, indicating reduced left ventricular function across the cohort.

The structural heart parameters included a mean LVEDD of 60.66 ± 5.38 mm, and left ventricular end-systolic diameter (LVESD) of 43.84 ± 5.58 mm. NT-proBNP, a biomarker for HF severity, averaged 8779.92 ± 3840.96 pg/mL. 

BP measurements indicated a mean systolic blood pressure (SBP) of 124.08 ± 15.97 mmHg and a diastolic blood pressure (DBP) of 70.34 ± 8.35 mmHg, indicating relatively controlled blood pressure levels in this population. HTN was present in 43.88% of patients, while ischemic heart disease (IHD) was observed in 88.77% of the cohort. DM and dyslipidemia were present in 38.77% and 55.10% of patients, respectively.

The average number of hospitalizations was 1.34 ± 1.03, and the mean duration of disease was 5.62 ± 1.08 years. 

The Hb levels were within normal range (14.3 ± 2.31 g/dL), and the creatinine clearance averaged 55.21 ± 20.56 mL/min. Arterial stiffness was assessed using the AIx, which averaged −3.70 ± 25.81%, and pulse wave velocity (PWV), with a mean value of 13.02 ± 1.79 m/s.

#### 3.1.2. General Characteristics of the Patients

Table 2 compares the clinical characteristics of male (*n* = 70) and female (*n* = 28) patients. There were no significant differences between the genders in most clinical parameters, including EF, AIx, PWV, LVEDD, LVESD, NT-proBNP, hospitalizations, or disease duration. However, dyslipidemia was significantly more common in males (*p* < 0.01), with 68.5% of males affected vs. 31.5% of females.

#### 3.1.3. Clinical Characteristics of HF Patients by HTN Status

Table 3 reveals significant differences between patients with and without HTN in several important cardiac and vascular parameters. Hypertensive patients exhibit notably distinct structural and functional characteristics compared to their non-hypertensive counterparts, particularly in terms of EF, LVEDD and end LVESD, and DBP.

As shown in Table 3, there were several significant differences between hypertensive (*n* = 43) and non-hypertensive (*n* = 55) HF patients. Hypertensive patients had significantly greater LVEDD, LVESD, and NT-proBNP levels, as well as higher SBP and DBP. Conversely, their EF was notably lower (26.00% ± 4.63 vs. 29.22% ± 5.31, *p* < 0.01), suggesting impaired cardiac function in hypertensive patients.

There were no significant differences in PWV (13.02 ± 1.74 vs. 12.97 ± 1.82, *p* = 0.88) or AIx (−2.09 ± 26.02 vs. −2.22 ± 25.94, *p* = 0.98) between the two groups.

#### 3.1.4. Clinical Characteristics of HF Patients by DM

Table 4 illustrates the differences between diabetic (*n* = 38) and non-diabetic (*n* = 60) HF patients. Diabetic patients had significantly lower EF (26.34% ± 5.35 vs. 28.73% ± 5.01, *p* = 0.02), higher negative AIx values (−10.74 ± 26.84 vs. 3.26 ± 23.85, *p* < 0.01), and higher PWV (13.62 ± 1.78 vs. 12.59 ± 1.67, *p* < 0.01), indicating a higher level of arterial stiffness.

Although there were differences in the NT-proBNP levels (9533.63 ± 4150.29 vs. 8166.11 ± 3608.60, *p* = 0.08) and SBP (120.66 ± 16.23 vs. 126.25 ± 15.55, *p* = 0.09), these were not statistically significant. Similarly, DBP and creatinine clearance were comparable between the two groups.

#### 3.1.5. Clinical Characteristics of HF Patients by Dyslipidemia Status

In Table 5, patients with dyslipidemia (*n* = 54) had significantly lower EF (26.72% ± 5.37 vs. 29.18% ± 4.75, *p* = 0.02), higher PWV (13.51 ± 1.77 vs. 12.43 ± 1.66, *p* < 0.01), and higher NT-proBNP levels (10,012.65 ± 4428.24 vs. 7267.02 ± 2204.54, *p* < 0.01) compared to patients without dyslipidemia (*n* = 44). Additionally, patients with dyslipidemia were older on average (61.81 ± 6.32 years vs. 59.23 ± 5.99 years, *p* = 0.04).

The BP levels were significantly lower in dyslipidemic patients, with SBP at 116.85 ± 14.50 mmHg compared to 132.95 ± 13.03 mmHg (*p* < 0.01) and DBP at 67.81 ± 7.00 mmHg compared to 73.43 ± 8.90 mmHg (*p* < 0.01).

No significant differences in creatinine clearance or disease duration were observed.

In the evaluation of arterial stiffness indicators among dyslipidemic patients undergoing statin therapy with optimal serum cholesterol levels, statistically significant differences were found, with patients with controlled cholesterol levels showing lower AIx (2.65 ± 25.66 vs. −27.80 ± 19.71, *p* < 0.01) and PWV (12.97 ± 1.76 vs. 14.13 ± 1.58, *p* < 0.01) compared to those with pathological cholesterol levels. These findings are illustrated in Table 6.

### 3.2. Analysis of the Clinical, Biochemical, Echocardiographic Parameters and Arterial Stiffness Indicators in Hospitalized Patients with HF Due to Acutely Decompensated HF

#### 3.2.1. Distribution of Cardiovascular Risk Factors by Gender in Patients with Chronic and Acutely Decompensated HF

Table 7 provides insights into the gender distribution and cardiovascular risk factors among patients with chronic and acutely decompensated HF. Notably, 83.02% of patients with chronic HF are male, suggesting that male patients may experience lower rates of acutely decompensation compared to females (*p* < 0.01). HTN is prevalent in both groups at 39.62%, while chronic illness affects 86.79% of all patients.

DM shows a stable prevalence of around 39% across both groups. However, dyslipidemia is more pronounced in the decompensation group, with 66.67% of those patients affected.

#### 3.2.2. Distribution of Arterial Stiffness Indicators and Clinical Parameters in Patients with Acutely Decompensated HF

Based on the findings presented in Table 8, the Pearson correlation analysis revealed a strong negative correlation between PWV and EF and a moderate positive correlation between AIx and EF in patients hospitalized due to acutely decompensated HF. The inclusion criteria specified that all patients were diagnosed with HF and either exhibited clinical signs of acutely decompensation HF or were managed as stable HF cases. Patients were categorized as either hospitalized for acutely decompensated HF or managed on an outpatient basis. Acutely decompensated HF was defined by a worsening clinical status requiring hospital admission, including elevated NT-proBNP levels, symptoms of congestion, and echocardiographic findings of deteriorating EF or increased ventricular dimensions.

The patient cohort (*n* = 92) comprised 53 individuals with acutely decompensated HF and 39 patients with chronic HF. Among the acutely decompensated HF group, 45 were hospitalized during the follow-up period. Table 8 illustrates the correlations between the arterial stiffness parameters and clinical variables within the acutely decompensated HF subgroup. The data suggest that higher PWV is linked to lower EF, while AIx tends to increase as EF increases. This indicates that arterial stiffness, as measured by PWV and AIx, is inversely related to heart function.

#### 3.2.3. Relationship Between EF and Arterial Stiffness Indicators (PWV and AIx)

**Figure 1:**
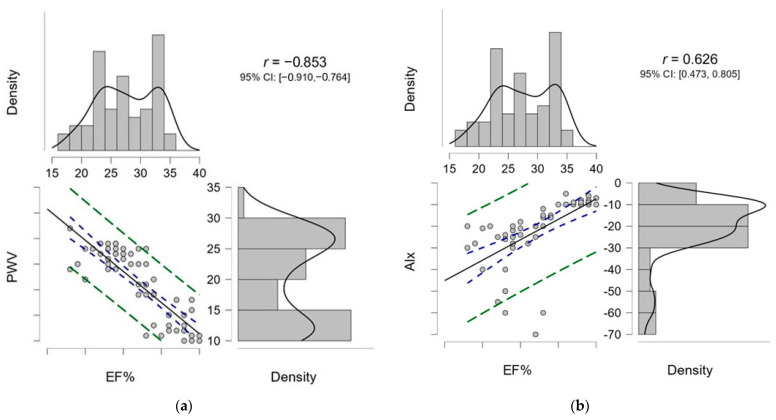
Correlation of the arterial stiffness markers (PWV and AIx) with EF in hospitalized patients (*n* = 59). (**a**) Correlation between PWV and EF. (**b**) Correlation between AIx and EF.

The relationship between EF and PWV was assessed using Pearson’s correlation coefficient in Figure 1a. A strong negative correlation was observed, with a correlation coefficient of r = −0.853, indicating a significant inverse relationship between the two variables. Specifically, higher EF% was associated with lower PWV, suggesting that, as heart function improves, arterial stiffness decreases. The 95% confidence interval (CI) for this correlation was [−0.910,−0.764], which does not include zero, further confirming that this relationship is statistically significant and unlikely to have occurred by chance. The regression line (blue) and the CI (green dashed lines) highlight this relationship, while the accompanying histograms and density curves depict the distributions of EF% and PWV.

In Figure 1b, the relationship between EF% and the AIx was also evaluated using Pearson’s correlation coefficient. A moderate positive correlation was found, with r = 0.626, indicating that, as EF% increases, AIx tends to increase as well. However, this relationship was less pronounced compared to the correlation between EF% and PWV. The 95% CI for this correlation was [0.473,0.805], which does not include zero, supporting the statistical significance of the positive relationship and suggesting that the observed association is unlikely to be due to random chance. The regression line (blue) demonstrates the relationship between EF% and AIx, with the green dashed lines representing the CI. The histograms and density curves on the margins depict the distributions of EF% and AIx.

#### 3.2.4. Comparative Analysis of Re-Hospitalized and Non-Hospitalized Patients

According to Table 9, patients who were re-hospitalized due to HF exhibited a significantly lower EF (*p* < 0.01), higher NT-proBNP levels (*p* < 0.01), and a greater LVESD (*p* < 0.01) compared to those who attended follow-up visits as outpatients.

The values of PWV and AIx demonstrated statistically significant differences between re-hospitalized and non-hospitalized patients, with PWV measuring 13.71 ± 1.76 in re-hospitalized patients compared to 12.38 ± 1.55 in their non-hospitalized counterparts (*p* < 0.01). Similarly, the AIx values were −15.35 ± 27.79 at re-hospitalized patients vs. 9.03 ± 17.72 at non-hospitalized.

The duration of illness was longer in re-hospitalized patients compared to those with chronic HF, with values of 5.85 ± 1.00 years vs. 5.43 ± 1.13 years (*p* = 0.05).

#### 3.2.5. Results of Multiple Cox Regression Analysis for Re-Hospitalization Risk Factors

Table 10 summarizes the findings of a multiple Cox regression analysis, identifying clinical and demographic variables that may influence the risk of re-hospitalization in HF patients. The results are as follows:

- Age (HR = 1.05): Each 1-year increase in age is associated with a 5% increase in the hazard of re-hospitalization, though this result is not statistically significant (*p* = 0.29).
- EF (HR = 0.92): A 1% decrease in EF is associated with an 8% increase in the hazard of re-hospitalization, and this is statistically significant (*p* < 0.01).
- AIx (HR = 1.03): A 1% increase in the AIx is associated with a 3% increase in the hazard of re-hospitalization, which is statistically significant (*p* = 0.03).
- PWV (HR = 1.15): For each 1 m/s increase in PWV, the hazard of re-hospitalization increases by 15%, and this is statistically significant (*p* = 0.02).
- LVEDD (HR = 1.02): A 1 mm increase in LVEDD is associated with a 2% increase in re-hospitalization risk, but this result is not statistically significant (*p* = 0.35).
- LVESD (HR = 1.06): A 1 mm increase in LVESD is associated with a 6% increase in re-hospitalization risk, and this is borderline statistically significant (*p* = 0.05).
- NT-proBNP (HR = 1.0001): Each unit increase in NT-proBNP is associated with a very small but statistically significant increase in re-hospitalization risk (*p* < 0.01).
- SBP (HR = 0.98): A 1 mmHg decrease in SBP is associated with a 2% decrease in the hazard of re-hospitalization, but this result is not statistically significant (*p* = 0.34).
- DBP (HR = 1.01): A 1 mmHg increase in DBP is associated with a 1% increase in the hazard of re-hospitalization, though this is not statistically significant (*p* = 0.48).
- Creatinine Clearance (HR = 0.98): A 1 mL/min decrease in creatinine clearance is associated with a 2% increase in re-hospitalization risk, and this result is statistically significant (*p* < 0.01).
- Hb (HR = 1.01): A 1 g/dL increase in hemoglobin is associated with a 1% increase in the hazard of re-hospitalization, but this result is not statistically significant (*p* = 0.63).
- Duration of Disease (HR = 1.03): For each additional year of disease duration, the hazard of re-hospitalization increases by 3%, but this result is not statistically significant (*p* = 0.46).

#### 3.2.6. Age-Based Differences in Clinical and Arterial Stiffness Parameters 

When stratifying patients by age, it was observed that patients aged 60 years and older exhibited more severe clinical profiles compared to those younger than 60. Older patients generally had lower EF and higher NT-proBNP levels, indicating more advanced HF. These patients also demonstrated increased arterial stiffness, as reflected by higher PWV and AIx values. This suggests that aging is associated with a more pronounced deterioration in both cardiac function and vascular health, which could contribute to poorer prognosis and higher risk of adverse outcomes, including re-hospitalization.

Additionally, older patients had a higher prevalence of comorbid conditions such as HTN and DM, which further complicate their management and may contribute to their worse clinical outcomes. These findings emphasize the importance of tailored management strategies that account for both age and comorbidity burden in older HF patients.

## 4. Discussion

This study provides valuable insights into the relationships between clinical parameters, biochemical indicators, and arterial stiffness in hospitalized patients with acutely decompensated HF. The findings align closely with the established hypotheses, suggesting that specific clinical and echocardiographic metrics play a critical role in predicting hospitalization risk and overall patient status [31,32].

### 4.1. Primary Hypothesis Interpretation

This study demonstrated a strong negative correlation between EF and PWV, indicating that improved cardiac function is associated with reduced arterial stiffness. In contrast, a moderate positive correlation was observed between EF% and AIx, suggesting a less pronounced relationship. These results are consistent with previous studies indicating that decreased EF is associated with increased arterial stiffness, reflecting the deteriorating cardiovascular function in patients with HF [33,34]. The contrasting relationships highlight the utility of PWV as a more sensitive marker of arterial stiffness compared to AIx when assessing the HF severity. Both PWV and AIx may serve as valuable biomarkers to evaluate disease progression and guide treatment strategies in HF management [22,29,35].

### 4.2. Comparative Hypothesis Findings

Re-hospitalized patients exhibited significantly worse clinical and biochemical parameters compared to non-hospitalized patients. These included lower EF%, elevated NT-proBNP levels, and increased PWV, all of which reflect the advanced stage of HF and heightened arterial stiffness in these patients. Additionally, reduced SBP and DBP further underscore the hemodynamic instability often associated with acutely decompensated HF. These results corroborate findings from earlier studies that have linked elevated NT-proBNP with poor outcomes in HF patients [36,37]. The observed differences in PWV and AIx values emphasize the role of arterial stiffness as a key factor in HF progression, supporting their utility as prognostic biomarkers. These distinctions highlight the importance of early detection and targeted interventions for patients at risk of acutely decompensated HF to prevent re-hospitalization and improve outcomes [38,39].

### 4.3. Risk Factor Hypothesis Analysis

The analysis revealed a significant difference in the prevalence of cardiovascular risk factors between patients with and without HF decompensation, particularly in the case of HTN and dyslipidemia. HTN was more prevalent in patients with acutely decompensated HF, and dyslipidemia was notably more common in the chronic HF group as well. This is consistent with the existing literature suggesting that these risk factors exacerbate HF symptoms and contribute to acutely decompensated HF. Additionally, lifestyle risk factors such as sedentary behavior, poor diet, and smoking remain common among individuals with cardiovascular disease. These behaviors are well-established in accelerating HF progression, emphasizing the need for interventions aimed at modifying these risk factors in the management of HF [40,41,42]. These findings highlight the critical role of comprehensive cardiovascular risk assessment in managing HF patients. Tailoring preventive strategies to address both traditional risk factors (such as HTN and dyslipidemia) and modifiable lifestyle factors is essential for reducing the risk of acutely decompensated HF and improving patient outcomes [43,44,45,46].

### 4.4. Predictive Hypothesis Outcomes

Arterial stiffness indicators, particularly PWV and AIx, were identified as independent predictors of hospitalization even when adjusted for other clinical parameters. This finding supports the hypothesis and echoes earlier research emphasizing the prognostic value of arterial stiffness in HF management. The implications of these results are significant; monitoring arterial stiffness may provide clinicians with critical insights into patient prognosis and facilitate timely intervention to mitigate adverse outcomes [47,48].

### 4.5. Gender Distribution Hypothesis Insights

This study also highlights a notable gender distribution between patients with and without HF decompensation, with a higher percentage of males in the chronic HF group. In contrast, females were more prevalent in the acutely decompensated HF group. These findings suggest that gender may influence the likelihood of experiencing acutely decompensated HF, with males potentially having a lower risk of decompensation compared to females. Previous studies have suggested that males and females may experience different disease trajectories and outcomes in HF, with gender-specific factors influencing the progression and management of the condition. Understanding these gender-based differences can be crucial in developing tailored, gender-specific strategies for HF management and treatment [29,49].

### 4.6. Comorbidity Hypothesis Examination

The results showed that patients experiencing acutely decompensated HF had a higher prevalence of comorbidities, reinforcing the hypothesis that concurrent illnesses exacerbate HF severity. This aligns with the established knowledge that managing comorbid conditions is crucial for improving HF outcomes. The burden of these comorbidities necessitates a holistic approach to treatment, which incorporates strategies for managing multiple health issues concurrently [50,51,52].

### 4.7. Re-Hospitalization Risk Hypothesis: Arterial Stiffness, EF, and NT-proBNP as Predictors

The results of the multiple Cox regression analysis support the hypothesis that arterial stiffness indices (PWV and AIx), EF, and NT-proBNP levels are independent predictors of re-hospitalization risk in HF patients. These findings align with existing literature, which highlights these markers as critical determinants of adverse outcomes in HF. Arterial stiffness markers, such as PWV and AIx, have been strongly linked to poor prognosis in HF patients, including higher re-hospitalization rates [53,54]. The current study’s results reinforce this understanding, emphasizing the importance of arterial stiffness in assessing HF severity and predicting re-hospitalization risk. This supports the integration of arterial stiffness assessments in routine HF management to better stratify patients and manage their risks.

#### 4.7.1. Arterial Stiffness as a Predictor

Arterial stiffness, measured by PWV and AIx, has consistently been shown to predict cardiovascular events, including re-hospitalization in HF patients. In this study, each 1 m/s increase in PWV was associated with a 15% higher risk of re-hospitalization (HR = 1.15, *p* = 0.02), while a 1% increase in AIx was linked to a 3% higher risk (HR = 1.03, *p* = 0.03). These results are consistent with prior studies, which have demonstrated the predictive value of PWV and AIx in HF. Increased PWV reflects stiffer arteries and a greater afterload on the heart, worsening HF symptoms. Higher PWV has been associated with a greater likelihood of cardiovascular events and hospitalizations. Similarly, AIx, which reflects central arterial stiffness, has been linked to poorer outcomes in HF patients. Both markers contribute to increased cardiac workload and deteriorating heart function, underlining their importance in predicting re-hospitalization risk in HF patients [55,56].

#### 4.7.2. EF as a Predictor

EF is a widely used measure of HF severity, and in this study, a 1% decrease in EF was associated with an 8% increase in the risk of re-hospitalization (HR = 0.92, *p* < 0.01). This reinforces the well-established role of EF as a prognostic marker in HF. A reduced EF indicates impaired systolic function and is linked to worse clinical outcomes, including hospitalization. Previous studies have shown that lower EF correlates with an increased likelihood of re-hospitalization. As the heart’s ability to pump blood declines, patients become more prone to fluid retention, worsening symptoms, and hospitalization. These findings further support the use of EF as a key clinical parameter for predicting re-hospitalization risk in HF patients [57,58].

#### 4.7.3. NT-proBNP as a Prognostic Marker

NT-proBNP, a biomarker of cardiac stress, is widely used to assess HF severity. This study found that NT-proBNP levels were significantly associated with an increased risk of re-hospitalization, with each unit increase in NT-proBNP correlating with a small but statistically significant rise in re-hospitalization risk (HR = 1.0001, *p* < 0.01). Elevated NT-proBNP levels signal worsening HF and are linked to increased risks of adverse outcomes, such as hospitalization and mortality. These findings are consistent with prior research and highlight NT-proBNP’s role in reflecting myocardial stress and acutely decompensated HF, making it a critical tool for clinicians in risk stratification and disease management [59,60].

#### 4.7.4. Implications for Clinical Practice

The findings of this study have important implications for clinical practice, particularly in the management of HF patients. The identification of PWV, AIx, EF, and NT-proBNP as independent predictors of re-hospitalization highlights the importance of incorporating these parameters into routine clinical assessments.

1. Enhanced Risk Stratification: By monitoring PWV and AIx, which are indicators of arterial stiffness, alongside traditional measures like EF and NT-proBNP, clinicians can more accurately assess the risk of re-hospitalization. Arterial stiffness markers provide additional information about cardiovascular health that goes beyond standard measures, offering a more comprehensive view of a patient’s condition. This multi-parameter approach allows for earlier identification of high-risk patients, enabling proactive management and tailored interventions.
2. Predicting Outcomes and Guiding Treatment: Integrating arterial stiffness markers with EF and NT-proBNP levels into clinical practice provides valuable prognostic information. For example, a high PWV or AIx, coupled with low EF and elevated NT-proBNP, could indicate worsening cardiovascular function and the potential for decompensation. This can help clinicians adjust treatment plans, such as optimizing HF medications, initiating or intensifying therapies aimed at reducing arterial stiffness, or monitoring patients more closely.
3. Prevention of Re-hospitalizations: One of the primary goals in HF management is to reduce hospital readmissions. The ability to identify patients at higher risk for re-hospitalization based on the combination of PWV, AIx, EF, and NT-proBNP can lead to timely interventions, such as adjustments in medical therapy, more frequent follow-up visits, or enhanced patient education on lifestyle modifications. For instance, patients with elevated NT-proBNP levels and increased arterial stiffness could benefit from more aggressive management of blood pressure and fluid retention to prevent further decompensation.
4. Clinical Decision-Making and Personalized Care: This study highlights the potential for a more personalized approach to HF care. By incorporating these markers into decision-making processes, clinicians can tailor treatment strategies to the specific needs of individual patients. For example, patients with elevated PWV and AIx may benefit from interventions targeting vascular health, such as lifestyle changes, antihypertensive therapy, or medications that improve arterial compliance, alongside standard HF treatments. This personalized approach can improve outcomes and enhance the quality of life for HF patients.

In summary, the integration of arterial stiffness indicators, EF, and NT-proBNP into routine clinical practice can significantly enhance the management of HF patients. This approach not only improves risk prediction and outcome forecasting but also allows for more targeted, personalized treatment strategies, ultimately reducing the likelihood of re-hospitalizations and improving patient outcomes in HF care.

#### 4.7.5. Potential Interventions to Influence Arterial Stiffness and Improve Cardiac Function

Given the importance of arterial stiffness as a predictor of poor outcomes in HF, targeting this factor could improve cardiac function and overall prognosis. Several approaches to reduce arterial stiffness have shown promise:

1. Pharmacological Interventions: Medications such as ACE inhibitors, ARBs, beta-blockers, and statins can improve arterial compliance and reduce systolic blood pressure, which, in turn, helps manage HF and enhance cardiac output. These therapies also address underlying conditions like HTN and dyslipidemia, both of which contribute to arterial stiffness [61,62].
2. Lifestyle Modifications: Regular physical activity and weight management have been shown to reduce arterial stiffness and improve heart function in HF patients. Additionally, heart-healthy diets (e.g., Mediterranean diets) can further help manage blood pressure and reduce vascular rigidity [63,64].
3. Management of Comorbidities: Controlling HTN and DM is crucial in reducing arterial stiffness. Effective blood pressure management, along with proper glycemic control, can slow the progression of vascular stiffness, leading to better cardiovascular outcomes in HF patients [65,66].
4. Innovative Therapies: Enhancing endothelial function and reducing inflammation are emerging therapeutic strategies to address arterial stiffness in HF patients. Endothelial nitric oxide synthase (eNOS) activators, which increase nitric oxide production, may help reduce vascular stiffness by promoting vasodilation and improving endothelial health. Although still under investigation, this approach holds promise for future HF treatments. Additionally, targeting chronic inflammation with novel agents such as IL-1 inhibitors or TNF-alpha antagonists could help decrease arterial stiffness, benefiting both vascular and cardiac health in HF patients. These innovative therapies offer potential for improving long-term outcomes in HF management [67,68,69].

In conclusion, influencing arterial stiffness through a combination of pharmacological treatment, lifestyle changes, and management of comorbid conditions is a promising approach to improving cardiac function in patients with HF. By reducing arterial stiffness, it may be possible to reduce the afterload, enhance coronary perfusion, and optimize cardiac function, ultimately improving clinical outcomes and reducing the burden of HF [70,71].

### 4.8. Research Limitations

This study has several limitations that should be considered when interpreting the findings:

Sample Size and Generalizability: The sample size, while adequate for preliminary analysis, may limit the generalizability of the results. A larger and more diverse cohort would strengthen the findings and allow for more robust comparisons across various demographics and clinical presentations of HF.

Cross-Sectional Design: The cross-sectional nature of this study restricts the ability to establish causal relationships between the assessed clinical parameters and hospitalization risk. Longitudinal studies would provide a clearer understanding of how changes in EF, NT-proBNP levels, and arterial stiffness indicators over time impact clinical outcomes.

Exclusion Criteria: The exclusion of patients with certain comorbidities, such as advanced renal failure, atrial fibrillation, and other significant cardiovascular conditions, may limit the applicability of the findings to the broader HF population. Future studies should consider including a wider range of patient profiles to better capture the complexities of HF management.

Single-Center Study: Conducting the research at a single institution may introduce biases related to specific clinical practices and patient demographics. Multi-center studies could enhance the external validity of the results by providing insights from a broader patient population.

Measurement Variability: Variability in the measurement techniques for arterial stiffness and other clinical parameters may impact the reliability of the findings. Standardizing measurement protocols and ensuring consistency across assessments would improve the accuracy and reproducibility of the results.

Potential Confounding Factors: While efforts were made to control for known confounding factors, there may still be unmeasured variables—such as patient adherence to treatment, lifestyle factors, or psychosocial influences—that could affect the outcomes. Future research should aim to account for these additional factors to provide a more comprehensive understanding of the relationships involved.

Acknowledging these limitations is crucial for interpreting the findings accurately and for guiding future research aimed at improving patient outcomes in HF management.

### 4.9. Future Research Directions

These findings pave the way for future research aimed at further elucidating the mechanisms linking arterial stiffness to HF progression. Longitudinal studies investigating the impact of interventions targeting arterial stiffness on HF outcomes are warranted. Additionally, exploring gender differences in HF presentations and responses to treatment could enhance personalized care strategies. Overall, this study contributes to the growing body of evidence highlighting the importance of comprehensive clinical assessment in the management of HF and the potential for improving patient outcomes through targeted monitoring of arterial stiffness and cardiovascular risk factors.

## 5. Conclusions

This study revealed several important insights into the relationship between heart function, arterial stiffness, and patient outcomes in HF. The key findings include a strong negative correlation between EF and PWV, indicating that improved cardiac function is associated with reduced arterial stiffness. Conversely, a moderate positive correlation was observed between EF% and AIx, suggesting a less pronounced relationship between heart function and arterial stiffness in these parameters. These findings emphasize the importance of PWV and AIx as potential biomarkers for assessing the severity HF and for monitoring the changes in arterial health over time. 

Furthermore, the study highlighted that re-hospitalized patients exhibited notably worse clinical profiles compared to their non-hospitalized counterparts. This finding emphasizes the critical importance of the early identification of high-risk patients, particularly those with worsened EF, elevated NT-proBNP levels, and increased arterial stiffness. Timely interventions in this vulnerable group could help prevent hospital readmissions and improve long-term health outcomes. 

The findings also demonstrate that cardiovascular risk factors, including HTN and dyslipidemia, are prevalent in patients experiencing acutely decompensated HF. This highlights the need for comprehensive management strategies that address both HF and its comorbidities. Effective control of these risk factors, alongside monitoring arterial stiffness, may improve the overall prognosis and reduce the burden of HF.

This study has important clinical implications. By integrating arterial stiffness measurements such as PWV and AIx into routine clinical assessments, healthcare providers can better stratify the risk and optimize individualized treatment plans for patients with HF. This approach not only allows for more precise management of HF but also enhances our ability to prevent re-hospitalizations and optimize long-term care. 

Additionally, this study underscores the significant role of arterial stiffness in HF, highlighting the potential for targeted interventions aimed at improving arterial health to enhance cardiac function. Interventions such as pharmacological treatments (e.g., ACE inhibitors and beta-blockers), lifestyle modifications (e.g., exercise and diet), and effective management of comorbidities (e.g., HTN and DM) can influence arterial stiffness and, in turn, improve cardiac performance.

Furthermore, patients aged 60 years and older were found to have more severe clinical profiles, including lower EF, higher NT-proBNP levels, and greater arterial stiffness, which may contribute to their higher risk of re-hospitalization. These findings highlight the need for age-specific management strategies in HF patients, with a focus on improving both cardiac and vascular health, particularly in older individuals.

In summary, this study contributes valuable insights into the multifaceted nature of HF and the interrelationship between clinical metrics and patient outcomes. It highlights the potential of targeted interventions focused on improving arterial stiffness and managing cardiovascular risk factors to enhance care for patients with acutely decompensated HF. The identification of PWV and AIx as independent predictors of re-hospitalization, combined with the emphasis on managing cardiovascular risk factors, highlights key areas for improving patient care. Future research should further investigate the therapeutic potential of interventions aimed at reducing arterial stiffness and controlling comorbidities. Understanding the mechanisms behind these associations will be crucial for refining treatment strategies and enhancing prognostic accuracy in the management of HF.

## Figures and Tables

**Table 1:** Clinical characteristics of patients.

Parameter	Mean ± Standard Deviation (*n* = 98)
Age, years	61.00 ± 6.27
Gender (*n*, %)	Male: 70 (71.43%) Female: 28 (28.5%)
EF, %	27.83 ± 5.22
LVEDD, mm	60.66 ± 5.38
LVESD, mm	43.84 ± 5.58
NT-proBNP, pg/mL	8779.92 ± 3840.96
SBP, mmHg	124.08 ± 15.97
DBP, mmHg	70.34 ± 8.35
HTN (*n*, %)	43(43.88%)
IHD (*n*, %)	87(88.77%)
DM (*n*, %)	38 (38.77%)
Dyslipidemia (*n*, %)	54 (55.10%)
Numbers of spitalization	1.34 ± 1.03
Duration of Disease, years	5.62 ± 1.08
Hb, g/dL	14.3 ± 2.31
Creatinine Clearance, mL/min	55.21 ± 20.56
AIx, %	−3.70 ± 25.81
PWV, m/s	13.02 ± 1.79

Gender (*n*, %)—gender distribution (*n* = number of patients, % = percentage); EF, %—ejection fraction (%); LVEDD, mm—left ventricular end-diastolic diameter (millimeter); LVESD, mm—left ventricular end-systolic diameter (millimeter); NT-proBNP, pg/mL—N-terminal Pro B-type natriuretic peptide (picograms per milliliter); SBP, mmHg—systolic blood pressure (millimeter of mercury); DBP, mmHg—diastolic blood pressure (millimeter of mercury); HTN (*n*, %)—hypertension (number of patients, percentage); IHD (*n*, %)—ischemic heart disease (number of patients, percentage); DM (*n*, %)—diabetes mellitus (number of patients, percentage); Dyslipidemia (*n*, %)—dyslipidemia (*n* = number, % = percentage); Hb, g/dL—hemoglobin (grams per deciliter); Creatinine Clearance, mL/min—creatinine clearance (milliliter per minute); AIx, %—augmentation index (%); PWV, m/s—pulse wave velocity (meters per second).

**Table 2:** Comparison of the clinical parameters between male and female patients.

Parameter	Male (*n* = 70)	Female (*n* = 28)	*p*-Value
EF, %	28.01 ± 5.33	27.36 ± 5.00	0.58
AIx, %	−0.98 ± 25.56	−10.52 ± 25.60	0.10
PWV, m/s	12.91 ± 1.79	13.31 ± 1.78	0.31
LVEDD, mm	60.41 ± 4.88	61.31 ± 6.55	0.46
LVESD, mm	43.52 ± 5.14	44.66 ± 6.60	0.36
NT-proBNP, pg/mL	8552.93 ± 3885.57	9347.39 ± 3735.11	0.36
Number of Hospitalizations	1.33 ± 1.00	1.39 ± 1.13	0.78
SBP, mmHg	125.11 ± 15.51	121.50 ± 17.07	0.31
DBP, mmHg	70.83 ± 8.65	69.11 ± 7.58	0.36
Duration of Disease, years	5.45 ± 1.23	6.00 ± 1.78	0.26
HTN (*n*, %)	30 (42.86%)	13 (46.43%)	0.10
IHD (*n*, %)	60 (85.71%)	27 (96.43%)	0.92
DM (*n*, %)	25 (35.71%)	13 (46.43%)	0.07
Dyslipidemia (*n*, %)	37 (68.5%)	17 (31.5%)	<0.01
Creatinine Clearance, mL/min	58.67 ± 16.78	55.21 ± 20.56	0.69

EF, %—Ejection Fraction (%); AIx, %—Augmentation Index (%); PWV, m/s—Pulse Wave Velocity (meters per second); LVEDD, mm—Left Ventricular End-Diastolic Diameter (millimeter); LVESD, mm—Left Ventricular End-Systolic Diameter (millimeter); NT-proBNP, pg/mL—N-Terminal Pro B-Type Natriuretic Peptide (picograms per milliliter); SBP, mmHg—Systolic Blood Pressure (millimeter of mercury); DBP, mmHg—Diastolic Blood Pressure (millimeter of mercury); HTN (*n*, %)—Hypertension (number of patients, percentage); IHD (*n*, %)—Ischemic Heart Disease (number of patients, percentage); DM (*n*, %)—Diabetes Mellitus (number of patients, percentage); Creatinine Clearance, mL/min—Creatinine Clearance (milliliter per minute).

**Table 3:** Comparison of the clinical parameters between patients with and without HTN.

Parameter	Patients with HTN (*n* = 43)	Patients Without HTN (*n* = 55)	*p*-Value
Age, years	59.84 ± 6.17	61.29 ± 6.34	0.25
EF, %	26.00 ± 4.63	29.22 ± 5.31	<0.01
AIx, %	−2.09 ± 26.02	−2.22 ± 25.94	0.98
PWV, m/s	13.02 ± 1.74	12.97 ± 1.82	0.88
LVEDD, mm	62.10 ± 5.87	59.54 ± 4.73	0.01
LVESD, mm	46.16 ± 5.57	42.03 ± 4.93	<0.01
NT-proBNP, pg/mL	8844.93 ± 3773.62	8580.24 ± 3965.94	0.73
SBP, mmHg	120.56 ± 14.54	126.84 ± 16.61	0.05
DBP, mmHg	67.98 ± 5.96	72.18 ± 9.48	0.01
Creatinine Clearance, mL/min	58.78 ± 18.16	56.83 ± 17.82	0.59
Hb, g/dL	13.92 ± 2.54	14.24 ± 2.30	0.51
Duration of Disease, years	5.54 ± 1.08	5.69 ± 1.10	0.49

EF, %—Ejection Fraction (%); AIx, %—Augmentation Index (%); PWV, m/s—Pulse Wave Velocity (meters per second); LVEDD, mm—Left Ventricular End-Diastolic Diameter (millimeter); LVESD, mm—Left Ventricular End-Systolic Diameter (millimeter); NT-proBNP, pg/mL—N-Terminal Pro B-Type Natriuretic Peptide (picograms per milliliter); SBP, mmHg—Systolic Blood Pressure (millimeter of mercury); DBP, mmHg—Diastolic Blood Pressure (millimeter of mercury); Creatinine Clearance, mL/min—Creatinine Clearance (milliliter per minute); Hb, g/dL—Hemoglobin (grams per deciliter).

**Table 4:** Comparison of the clinical parameters between patients with and without DM.

Parameter	Patients with DM (*n* = 38)	Patients Without DM (*n* = 60)	*p*-Value
Age, years	59.63 ± 6.63	61.30 ± 6.01	0.20
EF, %	26.34 ± 5.35	28.73 ± 5.01	0.02
AIx, %	−10.74 ± 26.84	3.26 ± 23.85	<0.01
PWV, m/s	13.62 ± 1.78	12.59 ± 1.67	<0.01
LVEDD, mm	60.84 ± 6.38	60.55 ± 4.71	0.80
LVESD, mm	44.44 ± 6.22	43.46 ± 5.17	0.40
NT-proBNP, pg/mL	9533.63 ± 4150.29	8166.11 ± 3608.60	0.08
SBP, mmHg	120.66 ± 16.23	126.25 ± 15.55	0.09
DBP, mmHg	70.58 ± 9.21	70.18 ± 7.84	0.82
Creatinine Clearance, mL/min	56.03 ± 17.76	58.73 ± 18.06	0.46
Hb, g/dL	14.09 ± 2.36	14.11 ± 2.45	0.96
Duration of Disease, years	5.77 ± 1.10	5.52 ± 1.07	0.28

EF, %—Ejection Fraction (%); AIx, %—Augmentation Index (%); PWV, m/s—Pulse Wave Velocity (meters per second); LVEDD, mm—Left Ventricular End-Diastolic Diameter (millimeter); LVESD, mm—Left Ventricular End-Systolic Diameter (millimeter); NT-proBNP, pg/mL—N-Terminal Pro B-Type Natriuretic Peptide (picograms per milliliter); SBP, mmHg—Systolic Blood Pressure (millimeter of mercury); DBP, mmHg—Diastolic Blood Pressure (millimeter of mercury); Creatinine Clearance, mL/min—Creatinine Clearance (milliliter per minute); Hb, g/dL—Hemoglobin (grams per deciliter).

**Table 5:** Comparison of the clinical parameters between patients with and without dyslipidemia.

Parameter	Patients with Dyslipidemia (*n* = 54)	Patients Without Dyslipidemia (*n* = 44)	*p*-Value
Age, years	61.81 ± 6.32	59.23 ± 5.99	0.04
EF, %	26.72 ± 5.37	29.18 ± 4.75	0.02
AIx, %	−11.45 ± 27.54	7.87 ± 20.12	<0.01
PWV, m/s	13.51 ± 1.77	12.43 ± 1.66	<0.01
LVEDD, mm	60.57 ± 6.52	60.77 ± 3.62	0.85
LVESD, mm	44.44 ± 6.27	43.11 ± 4.58	0.24
NT-proBNP, pg/mL	10,012.65 ± 4428.24	7267.02 ± 2204.54	<0.01
SBP, mmHg	116.85 ± 14.50	132.95 ± 13.03	<0.01
DBP, mmHg	67.81 ± 7.00	73.43 ± 8.90	<0.01
Creatinine Clearance, mL/min	54.44 ± 17.75	61.67 ± 17.47	0.05
Hb, g/dL	14.21 ± 2.28	13.97 ± 2.56	0.62
Duration of Disease, years	5.23 ± 1.10	5.63 ± 1.12	0.85

EF, %—Ejection Fraction (%); AIx, %—Augmentation Index (%); PWV, m/s—Pulse Wave Velocity (meters per second); LVEDD, mm—Left Ventricular End-Diastolic Diameter (millimeter); LVESD, mm—Left Ventricular End-Systolic Diameter (millimeter); NT-proBNP, pg/mL—N-Terminal Pro B-Type Natriuretic Peptide (picograms per milliliter); SBP, mmHg—Systolic Blood Pressure (millimeter of mercury); DBP, mmHg—Diastolic Blood Pressure (millimeter of mercury); Creatinine Clearance, mL/min—Creatinine Clearance (milliliter per minute); Hb, g/dL—Hemoglobin (grams per deciliter).

**Table 6:** Values of arterial stiffness indicators in patients with pathological vs. controlled cholesterol levels.

Prameter	Patients with Pathological Cholesterol Levels (*n* = 10)	Patients with Controlled Cholesterol Levels (*n* = 44)	*p*-Value
AIx, %	−27.80 ± 19.71	2.65 ± 25.66	<0.01
PWV, m/s	14.13 ± 1.58	12.97 ± 1.76	<0.01

AIx, %—augmentation index (%); PWV, m/s—pulse wave velocity (meters per second).

**Table 7:** Baseline characteristics of patients with chronic and acutely decompensated HF.

Variables		Chronic HF (*n* = 53)	*p*	Acutely Decompensated HF (*n* = 45)	*p*
Gender (*n*, %)	Male	44 (83.02%)	<0.01	26 (57.78%)	0.06
	Female	9 (16.98%)	<0.01	19 (42.22%)	0.06
HTN (*n*, %)		21 (39.62%)	0.03	22 (48.89%)	0.11
Chronic Illness (*n*, %)		46 (86.79%)	<0.01	41 (91.11%)	<0.01
DM (*n*, %)		21 (39.62%)	0.03	17 (37.78%)	0.03
Dyslipidemia (*n*, %)		24 (45.28%)	0.08	30 (66.67%)	<0.01

HTN (*n*, %)—hypertension (number, percentage); DM (*n*, %)—diabetes mellitus (number, percentage); Dyslipidemia (*n*, %)—dyslipidemia (*n* = number, % = percentage).

**Table 8:** Correlations of the arterial stiffness indicators with the clinical parameters in patients with acutely decompensated HF (*n* = 45).

Parameter	PWV	*p*	AIx	*p*
Age, years	−0.0102	0.92	−0.0004	0.99
EF, %	−0.853	<0.01	0.626	<0.01
LVEDD, mm	0.0631	0.53	−0.2233	0.02
LVESD, mm	0.1635	0.10	−0.3184	<0.01
NT-proBNP, pg/mL	0.5835	<0.01	−0.6858	<0.01
SBP, mmHg	−0.3371	<0.01	0.4779	<0.01
DBP, mmHg	−0.1078	0.29	0.2953	<0.01
Creatinine Clearance, mL/min	−0.2144	0.03	0.2493	0.01
Hb, g/dL	−0.1575	0.12	0.0869	0.39
Duration of Disease, years	0.0483	0.63	−0.3071	<0.01

EF, %—Ejection Fraction (%); LVEDD, mm—Left Ventricular End-Diastolic Diameter (millimeter); LVESD, mm—Left Ventricular End-Systolic Diameter (millimeter); NT-proBNP, pg/mL—N-Terminal Pro B-Type Natriuretic Peptide (picograms per milliliter); SBP, mmHg—Systolic Blood Pressure (millimeter of mercury); DBP, mmHg—Diastolic Blood Pressure (millimeter of mercury); Creatinine Clearance, mL/min—Creatinine Clearance (milliliter per minute); Hb, g/dL—Hemoglobin (grams per deciliter).

**Table 9:** Characteristics of re-hospitalized vs. non-hospitalized patients.

Parameter	Re-Hospitalized (*n* = 59)	Non-Hospitalized (*n* = 39)	*p*-Value
Age, years	61.36 ± 6.63	60.03 ± 5.95	0.29
EF, %	24.78 ± 5.01	30.38 ± 3.94	<0.01
AIx, %	−15.35 ± 27.79	9.03 ± 17.72	<0.01
PWV, m/s	13.71 ± 1.76	12.38 ± 1.55	<0.01
LVEDD, mm	61.22 ± 6.84	60.19 ± 3.75	0.35
LVESD, mm	45.50 ± 6.16	42.43 ± 4.66	<0.01
NT-proBNP, pg/mL	11,764.44 ± 3737.09	6091.42 ± 994.17	<0.01
SBP, mmHg	116.02 ± 16.56	130.92 ± 11.82	<0.01
DBP, mmHg	67.42 ± 6.82	72.81 ± 8.79	<0.01
Creatinine Clearance, mL/min	52.46 ± 18.18	62.12 ± 16.57	<0.01
Hb, g/dL	14.21 ± 2.54	14.01 ± 2.30	0.69
Duration of Disease, years	5.85 ± 1.00	5.43 ± 1.13	0.05

EF, %—Ejection Fraction (%); AIx, %—Augmentation Index (%); PWV, m/s—Pulse Wave Velocity (meters per second); LVEDD, mm—Left Ventricular End-Diastolic Diameter (millimeter); LVESD, mm—Left Ventricular End-Systolic Diameter (millimeter); NT-proBNP, pg/mL—N-Terminal Pro B-Type Natriuretic Peptide (picograms per milliliter); SBP, mmHg—Systolic Blood Pressure (millimeter of mercury); DBP, mmHg—Diastolic Blood Pressure (millimeter of mercury); Creatinine Clearance, mL/min—Creatinine Clearance (milliliter per minute); Hb, g/dL—Hemoglobin (grams per deciliter).

**Table 10:** Predictors of re-hospitalization in HF patients (*n* = 59).

Variable	HR	95% CI	*p*
Age, years	1.05	0.98–1.12	0.29
EF, %	0.92	0.85–0.98	<0.01
AIx, %	1.03	1.01–1.05	0.03
PWV, m/s	1.15	1.02–1.28	0.02
LVEDD, mm	1.02	0.98–1.06	0.35
LVESD, mm	1.06	1.00–1.12	0.05
NT-proBNP, pg/mL	1.0001	1.0000–1.0002	<0.01
SBP, mmHg	0.98	0.95–1.01	0.34
DBP, mmHg	1.01	0.98–1.05	0.48
Creatinine Clearance, mL/min	0.98	0.96–1.00	<0.01
Hb, g/dL	1.01	0.98–1.04	0.63
Duration of Disease, years	1.03	0.97–1.09	0.46

EF, %—Ejection Fraction (%); AIx, %—Augmentation Index (%); PWV, m/s—Pulse Wave Velocity (meters per second); LVEDD, mm—Left Ventricular End-Diastolic Diameter (millimeter); LVESD, mm—Left Ventricular End-Systolic Diameter (millimeter); NT-proBNP, pg/mL—N-Terminal Pro B-Type Natriuretic Peptide (picograms per milliliter); SBP, mmHg—Systolic Blood Pressure (millimeter of mercury); DBP, mmHg—Diastolic Blood Pressure (millimeter of mercury); Creatinine Clearance, mL/min—Creatinine Clearance (milliliter per minute); Hb, g/dL—Hemoglobin (grams per deciliter).

## Data Availability

The raw data supporting the conclusions of this article will be made available by the authors on request.
